# Lipid Nanoparticles and Their Hydrogel Composites for Drug Delivery: A Review

**DOI:** 10.3390/ph11040118

**Published:** 2018-11-01

**Authors:** Claire Desfrançois, Rachel Auzély, Isabelle Texier

**Affiliations:** 1 CEA LETI MlNATEC Campus, University Grenoble Alpes, F-38000 Grenoble, France; claire.desfrancois@cea.fr; 2 CERMAV-CNRS, University Grenoble Alpes, F-38000 Grenoble, France; rachel.auzely@cermav.cnrs.fr

**Keywords:** lipid nanoparticles, hydrogels, polysaccharides, solid lipid nanoparticles, nanostructured lipid carriers, chitosan, dextran, cellulose, alginate

## Abstract

Several drug delivery systems already exist for the encapsulation and subsequent release of lipophilic drugs that are well described in the scientific literature. Among these, lipid nanoparticles (LNP) have specifically come up for dermal, transdermal, mucosal, intramuscular and ocular drug administration routes in the last twenty years. However, for some of them (especially dermal, transdermal, mucosal), the LNP aqueous dispersions display unsuitable rheological properties. They therefore need to be processed as semi-solid formulations such as LNP-hydrogel composites to turn into versatile drug delivery systems able to provide precise spatial and temporal control of active ingredient release. In the present review, recent developments in the formulation of lipid nanoparticle-hydrogel composites are highlighted, including examples of successful encapsulation and release of lipophilic drugs through the skin, the eyes and by intramuscular injections. In relation to lipid nanoparticles, a specific emphasis has been put on the LNP key properties and how they influence their inclusion in the hydrogel. Polymer matrices include synthetic polymers such as poly(acrylic acid)-based materials, environment responsive (especially thermo-sensitive) polymers, and innovative polysaccharide-based hydrogels. The composite materials constitute smart, tunable drug delivery systems with a wide range of features, suitable for dermal, transdermal, and intramuscular controlled drug release.

## 1. Introduction

One of the biggest challenges in pharmaceutical technology nowadays remains the design and the complete characterization of a sustainable and targeted drug delivery system, specifically for poorly water-soluble or insoluble active compounds. In this context, topical administration of drugs, especially the control and determination of the exact amount of active compound that reaches different skin layers [1], is still a hurdle to be overcome.

In the last two decades, the interest in lipid-based formulations for dermal and transdermal drug delivery, as well as for other potential drug administration routes, has steadily grown [2,3]. The most recent developments have been focused on improving the low bioavailability of lipophilic drugs and controlling their release [4]. Solid lipid nanoparticles (SLN) and lipid nano-emulsions (LNE) were first developed in the early 1990s [5,6]. SLN and LNE are colloidal nanoparticles with a lipophilic core that is, respectively, in a solid state or in a liquid state at room temperature. SLN and LNE ingredients are, most of the time, non-toxic and highly biocompatible, thus have been approved by the FDA (Food and Drug Administration) and the EMA (European Medicines Agency) [7]. The lipophilic core is supposed to protect the active molecule entrapped inside from environmental (UV, pH) or physiological (immune system, enzymes) degradations while providing good solubility for the lipophilic drug and preventing the formation of aggregates. However, SLN and LNE can present some limitations concerning the stability of drug encapsulation. Whereas important drug leakage can be observed for liquid-core LNE [6], the crystalline core of SLN can induce phase demixing and drug expulsion [7]. A new generation of lipid nanoparticles (LNP) was therefore introduced in the 2000s [8]: nanostructured lipid carriers (NLC). Their core structure is composed of a blend of liquid and solid lipids, potentially able to entrap higher payloads of active molecules, while better controlling their release thanks to the blend of lipids [5]. 

The nanometer size of SLN, LNE and NLC is a big asset for targeted delivery purposes. For instance, their efficient uptake in tumor tissues thanks to the Enhanced Permeability and Retention Effect (EPR) has been demonstrated in different models [9,10]. However, for some administration routes, such as dermal, transdermal, ocular, and transmucosal, LNP aqueous dispersions are more efficient and easy to apply when they are supported by a semi-solid vehicle. That is why, since 2004 [11], hydrogel formulations combined with LNP have been being tested for these applications [12,13,14].

Hydrogels are defined as 3D networks of polymers that swell in water while retaining their physical integrity. The gelation phenomenon is accounted for by the physical or chemical cross-linking of polymer chains. In most chemically cross-linked hydrogels, the junction points are stable, permanent, covalent bonds. By contrast, in the case of physically cross-linked hydrogels, the networks are held together by molecular entanglements and/or secondary forces, such as ionic, hydrogen-bonding or hydrophobic interactions. Finally, two or more polymer networks can be interlaced on a polymer scale, creating interpenetrating polymer network hydrogels. Hydrogels also have tunable physical and mechanical properties for a wide range of applications in medicine. In this regard, they have already been used as drug delivery systems for several years, as they offer a convenient supporting matrix for active ingredients [15]. However, when it comes to dermal or transdermal delivery of lipophilic drugs, they quickly reach their limits, since lipophilic drugs are not well stabilized into the hydrophilic matrix, where they can precipitate as aggregates or be released in an uncontrolled burst.

**Figure 1:**
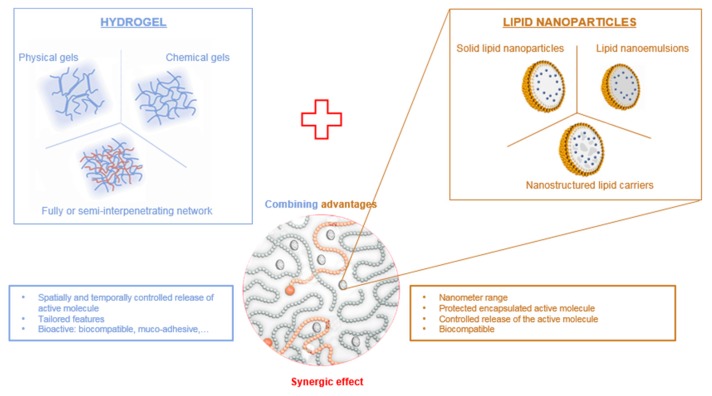
Combination of hydrogels and lipid nanoparticles.

Therefore, hydrogels can be advantageously combined with LNP to provide a double encapsulation strategy for the controlled release of lipophilic drugs (Figure 1). Lipophilic drugs can be efficiently loaded into lipid nanoparticles for which the lipid core provides a suitable matrix, LNP being themselves encapsulated inside the hydrogel scaffold. The double encapsulation provides an additional level of control over the spatial and temporal release of the drug, while benefiting from the biological properties of the hydrogel. In this review, we provide a comprehensive outlook on the combination of lipid nanoparticles and various types of hydrogels as drug delivery systems with an emphasis on polysaccharide-based hydrogels. 

## 2. Lipid Nanoparticles

**Figure 2:**
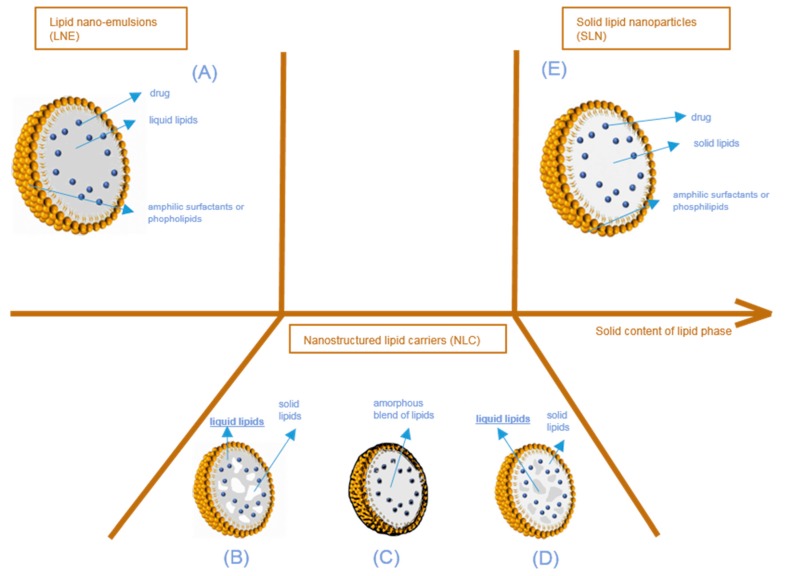
Structure of lipid nanoparticles according to the physical state of the lipid core, from lipid nano-emulsions (**A**) to solid lipid nanoparticles (**E**), through nanostructured lipid carriers (**B**–**D**). Different types of nanostructured lipid nanocarriers are possible according to the physical state of the blend of liquid and solid lipids composing the particle core (solid pockets in liquid (**B**), amorphous blend (**C**), liquid pockets in solid (**D**)).

Lipid nanoparticles (LNP) are colloidal lipophilic systems made of a lipid core stabilized in aqueous media by a single layer of surfactants (phospholipids, poly(ethylene glycol)-based (PEGylated) surfactants, etc.) (Figure 2). LNP thus display a lipophilic core and should not be confused with liposomes composed of an aqueous core stabilized by a surfactant bilayer. As mentioned above, three kinds of lipid nanoparticles are currently used as drug delivery systems: solid lipid nanoparticles (SLN), lipid nano-emulsions (LNE), and nanostructured lipid carriers (NLC). While all three types of LNP include lipids in their formulation, they can be differentiated by their core structure as described in Figure 2. Lipid nanoparticles used in drug delivery systems have an average size in the nanometer range. The majority of the ingredients entering the LNP composition are FDA and EMA approved, with the lipid composition remaining similar to lipophilic physiological molecules, while being adapted to fit the active molecule encapsulated. They are non-toxic and biodegradable. 

LNP are used as drug delivery systems, protecting and stabilizing hydrophobic active molecules in aqueous solutions. Their primary advantage is the improvement of the bioavailability of drugs, specifically lipophilic molecules that can be better solubilized in the lipid core than in aqueous environments. The lipophilic core of lipid nanoparticles entraps active ingredients, while the surfactant membrane, generally consisting solely or partially of phospholipids, ensures the stability of LNP in hydrophilic environment. LNP are particularly well suited for transdermal drug delivery. Indeed, lipid nanoparticles are known to create a mono-layer film on the skin, limiting water evaporation and improving skin hydration [16].

### 2.1. Composition and Structure 

Two parameters are usually used to quantify the active molecule content encapsulated in LNP. The first is entrapment efficiency (E_e_), defined as:

(1) $$E_{e}\;=\;\frac{W_{t}\;-\;W_{f}}{W_{t}\;}$$

where W_t_ is the total amount of active compound added and W_f_ is the free amount of active compound remaining in the supernatant after purification and separation from the LNP. Finally, it defines the ratio between the weight of active compound actually encapsulated in the LNP and the weight of the active compound added to the lipid phase for particle production.

The second parameter is the loading capacity (L_c_), defined as:

(2) $$L_{c}\;=\;\frac{W_{t}\;-\;W_{f}}{W_{t}\;-\;W_{f}\;+W_{l}\;}$$

where W_l_ is the weight of lipids in the formulation. L_c_ thus expresses the ratio between the weight of the active compound entrapped in the LNP and the weight of lipids.

E_e_ and L_c_ vary with the lipophilic properties of the active molecule, the composition and the ratio of the solid and liquid lipids, the phospholipid types and amount, and the production methods.

As described above, three types of lipid nanoparticles have been used as drug delivery systems since the beginning of the 2000s (Figure 2). The first one consists of lipid nano emulsions (LNE), the core of which is composed of liquid lipids (Figure 2A). Limited by the very fast diffusion of the drug from their lipid core and their very poor stability, LNE have not been extensively used in drug delivery applications, except for parenteral nutrition. In some cases, a solid shell can be designed to obtain lipid nano-capsules [17]. The second type consists of solid lipid nanoparticles (SLN) (Figure 2E), which differ from the third one, called nanostructured lipid carriers (NLC), in terms of the lipid core composition. Simply described, SLN only have a core with lipids in a solid state at room and body temperature. However, in NLC, the lipid core is a heterogeneous mixture (Figure 2B,D), or an amorphous phase (Figure 2C) composed of solid and liquid lipids.

SLN were the first model used to better understand drug encapsulation in lipid nanoparticles. They can be produced either by hot or cold homogenization techniques. In both cases, the molecule of interest is dissolved in the melted lipid phase. In hot homogenization, the aqueous solution containing the surfactant is mixed at the same high temperature, immediately followed by the homogenization of the pre-emulsion by high-pressure homogenization or ultrasonication. Ultrasonication, though efficient, is difficult to implement for large sample batches, and the presence of metal contaminants originating from the sonication tip can compromise emulsion quality. In cold homogenization, the lipid phase containing the drug is cooled down to solid state, ground to microparticles, and finally dispersed in a cold surfactant aqueous solution. Homogenization is also carried out at or below room temperature [7]. Cold homogenization can be preferable when the drug to be encapsulated is temperature-sensitive.

There are commonly three different models for describing the incorporation of active drugs into SLN [5]: homogeneous drug lipid matrix, drug-enriched core, and drug-enriched membrane. The type of system obtained depends on the formulation used (lipids, active compound, phospholipids, surfactants) and of the method of production (hot or cold homogenization) [5]. 

In the homogeneous SLN matrix, there is no phase separation between the active molecule and the lipid core. This is achieved when SLN are produced by hot homogenization and encapsulate highly lipophilic drugs. These types of particle provide a long and sustainable release of the compound, which can last several weeks [5]. 

However, drugs cannot always be solubilized with high payloads in a solid lipid matrix. During the cooling and solidification of the particle core, the concentration of the active drug in the remaining liquid lipid phase can keep increasing, leading to the crystallization of an active drug-enriched shell. This results in phase separation between the poorly drug-loaded core lipid phase and the active molecule, generally located at the particle surface. If the active molecule presents limited solubility in the melted lipids, this surface drug enrichment provides a fast drug release. This type of release kinetics may be highly desirable in transdermal drug delivery to improve the skin permeation of the active molecule thanks to the monolayer formed by the SLN [5]. In contrast, when the active drug phase starts cooling and crystallizing first, the shell is deprived of active compounds. The kinetics of drug release then fits Fick’s law of diffusion [5]. Of course, these models are ideal. Most of the time, SLN architectures are closer to complex hybrid models. 

In nanostructured lipid carriers (NLC) (Figure 2B–D), the core is composed of a mixture of liquid and solid lipids. Blending two types of lipids, very different in their physical states, creates imperfections (i.e., non-crystallization) in the lipid core. These imperfections provide additional spaces in which the active compound can fit, enabling a larger amount of drug to be loaded when compared to perfect crystalline structures. With NLC again, there are several architectural models that can be examined. 

The first situation is the multiple NLC type (Figure 2B,D). Separated, liquid at room and body temperature lipid pockets are entrapped in a solid lipid matrix (Figure 2D), or solid lipid matrix domains are encapsulated in a liquid core (Figure 2B). This happens when the concentration of liquid lipids in the solid lipids is higher than their solubility. When the lipid mixture cools down during the production process, phase separation occurs, creating liquid lipid droplets or crystalline zones of solid lipids. This type of NLC is particularly interesting for increasing the content of the active molecule when the latter is more soluble in either the selected oil or in the solid lipids. This is the case for the majority of NLC described in the literature [18]. 

The second possibility is that the lipid mixture can form an amorphous blend (Figure 2C). This situation happens when the lipid mixture does not recrystallize during the production process, but still forms a solid matrix at room and body temperature [19] (i.e., homogeneous, amorphous and very viscous). This situation improves the very short-term and long-term stability of NLC; as the recrystallization is limited, fewer active compounds will be expelled from the NLC. 

### 2.2. Stability of LNP: Physicochemical Properties

The LNP shelf-life stability is determined mainly by two different sets of parameters. The first is directly related to the particles’ physical properties (size, size distribution, zeta potential, crystallinity profile), whereas the second set of parameters evaluates the stability of the encapsulated ingredient over time. 

The average diameter and the size distribution of lipid nanoparticles are the most examined parameters during LNP studies, from the first development steps through to preclinical and clinical trials. They are known to affect body absorption, biodistribution and excretion of nano-objects [20].

Dynamic light scattering (DLS) is the most widely used technique to monitor these two parameters. DLS is fast and easy to implement, and is one of the only nanometer range-suitable analysis methods for which International Standard guidelines have been decreed and described by the NCL (Nanomedicine Characterization Laboratory) and the EUNCL (European Nanomedicine Characterisation Laboratory) [21]. DLS provides information regarding two parameters: the average hydrodynamic diameter of the nanoparticle population, and the polydispersity index (PDI). However, it is a low-resolution method that is not able to detect small aggregates and small changes in particle size distribution caused by physiological conditions. Similarly, it cannot be used to resolve the particle size distribution of polydisperse solutions [22]. 

Alternative methods exist, such as nanoparticle tracking analysis (NTA) and Field Flow Fractionation coupled with Multi Angle Light Scattering (FFF-MALS) [22]. NTA is based on the same principle as DLS, namely, light scattering and Brownian motion. The difference lies in the data analysis. In NTA, each single particle movement is tracked, with the software calculating the diffusion coefficient of individual nanoparticles. This allows differentiation between slightly different nanoparticles. It is thus a high-resolution method. Sometimes, the refractive index of the LNP mixture does not allow for accurate light-scattering measurements. 

A fractionation step prior to the analysis can greatly improve the quality of the obtained size distribution profile. These fractionation systems, such as asymmetric flow FFF (shortly named A4F) or size exclusion chromatography, are usually associated with detectors (DLS or MALS). The whole system can resolve very complicated samples, giving a very precise outlook on the properties, behavior and stability of nanoparticles in biologically relevant environment [22]. 

**Figure 3:**
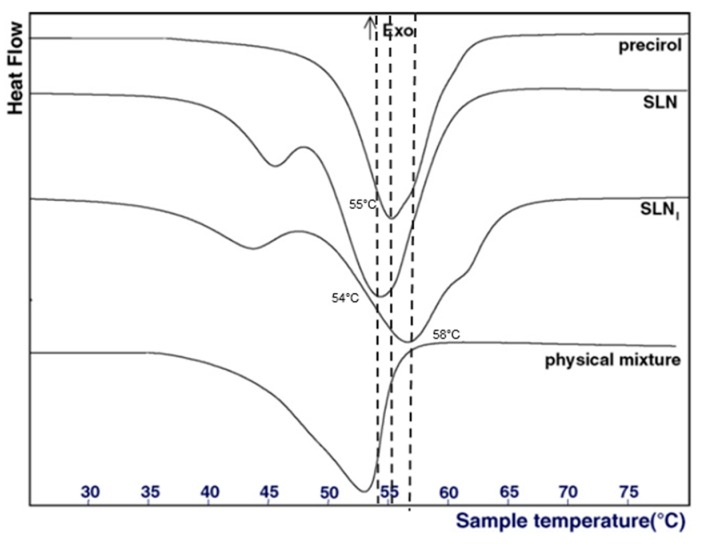
DSC profiles of pure precirol, empty SLN, ibuprofen-loaded SLN (SLN_i_) and a mixture of precirol and ibuprofen. Adapted from [23].

Concerning the analysis of the physical state of the lipid core, different techniques are available. Differential Scanning Calorimetry (DSC) is the most common method used to detect any changes in the crystallinity of LNP as a function of the temperature. In this regard, it generally helps to determine two parameters: (1) the physical status of encapsulated lipids (liquid, solid, or a mixture), and (2) the physical state of the loaded active molecule. By comparing the DSC profiles of bulk materials with those of nanoparticles, information about the physical state of the mixture of liquid and solid lipids can be deduced. Usually, a mixture of two lipids or a mixture of lipid and active molecules displays a lower melting temperature and a lower enthalpy of the melting peak than those of the pure compound [23]. For example, Casadei et al. described that the melting point of SLN encapsulating ibuprofen was 58 °C, whereas the melting point of pure ibuprofen was 76 °C (Figure 3), demonstrating interactions between the lipid core and the active molecule [23]. Furthermore, empty SLN display a melting point at 54 °C, whereas the melting point of pure Precirol (the solid lipid used in the formulation) was 55 °C [23]. This decrease was due to the interaction of Precirol with the surfactants, namely sodium cholate and Pluronic F68. 

Another method to characterize the physical state of the lipid core is X-ray Diffraction (XRD). XRD gives information on the crystallinity state of LNP lipid core, which can be compared to that of bulk ingredients entering the particle composition. Behbahani et al., compared XRD patterns of pure curcumin, bulk lipids entering the LNP composition, and of empty and loaded LNP [24]. They concluded that curcumin was completely dissolved in the lipid matrix and stabilized in amorphous form. The lower intensity of empty and loaded LNP patterns when compared to bulk lipid patterns also indicated the lower degree of crystallinity of these lipids in LNP. These results support the idea that the lipid matrix is less ordered in LNP [24]. 

To quantify and assess the stability of active ingredients entrapped inside the LNP core over the shelf-life time, the common analysis method is High-Performance Liquid Chromatography (HPLC) coupled with appropriate detectors. Small aliquots of the studied sample are regularly withdrawn, dialyzed or passed through ultrafiltration or solid phase extraction (SPE) to separate the free drug from the encapsulated drug, then LNP are destroyed with organic solvent and the final sample is injected into HPLC [19,25]. This method is substance-specific, offering the possibility to identify the substance thanks to the retention time, to quantify it thanks to pre-acquired calibration curves and to differentiate it from other compounds or impurities. This method can, however, be limited by the fractionation process required prior to the HPLC analysis; substances can remain adsorbed on the dialysis or filtration membrane depending on their surface charges, their sizes and the buffer used. Dilutions prior to separation and analysis can, moreover, provoke undesired drug release and introduce a bias in the result [22]. 

Compared to HPLC, Analytical Ultracentrifugation (AUC) is an alternative method, still in development. The AUC principle is to calculate the sedimentation coefficient of each particle to deduce its diameter and its molecular weight [22]. In a fast and easy way, AUC gives information on the particle size distribution profile and the free/bound weight ratio of the active molecule. It combines separation, concentration, and analysis steps in one experiment. 

Casadei et al. used ^1^H NMR (Nuclear Magnetic Resonance of protons) to quantify the ibuprofen content of SLN [23]. Without any further treatments before the measurements, they quantified the free ibuprofen contained in the aqueous phase of the formulation thanks to calibration with an internal standard. From the free ibuprofen weight, they were then able to deduce the drug encapsulation efficiency inside the nanoparticles (52 ± 3%) [23]. Prior studies showed that ibuprofen contained in the solid lipid core did not appear on the ^1^H NMR spectrum at room temperature [23]. However, this method is limited to the active molecule encapsulated in SLN. If the lipid core is not completely homogeneous, encapsulated ibuprofen may appear on the NMR spectrum. NMR can also be used to gain insight into the drug localization inside the LNP structure [6,19,26]. For instance, Delmas et al. used ^1^H NMR NOE spectra to gain information on Nile Red spatial proximity with NLC lipid core components. In this case, the ability of Nile Red to present environment-sensitive fluorescent properties confirmed the localization obtained by NMR, and allowed the quantitative analysis of dye distribution between particle core and surfactant shell [26]. 

### 2.3. Controlling Drug Release

One major reason for using LNP is their ability to control drug release kinetics. The drug delivery kinetics from LNP depends on their composition, the nature of the active molecule, and its localization inside the nanoparticle. The interactions between the active molecule and the liquid and solid lipids are especially crucial. In vitro tests have shown that LNP dispersions generally provide a prolonged release of loaded drugs [7]. The constraints of the in vivo environment can, however, require some adaptations to the design of LNP. To improve the blood half-life of the particles, PEG-based surfactants can be included as membrane ingredients. Adding PEG chains on the surface of the nanoparticle allows to prolong systemic circulation time and decrease immunogenicity [27,28]. 

While LNP provide stability and enhance the solubility of hydrophobic active molecules, drug release from LNP still, sometimes, displays a characteristic burst effect profile. This is especially the case when the active molecule is located both in the core and at the membrane of the lipid nanoparticle [5]. This specific kinetics can be desirable for transdermal delivery; it creates a mono-layer of the active ingredient on the skin, improving the drug permeation through the *stratum corneum* [29,30]. However, it is also often seen as undesirable because of its contribution to local or systemic drug toxicity and because it also reduces the blood half-time of the drug. It may lead to a poor delivery of the active ingredient and a poor therapeutic outcome. 

Moreover, when LNP are used for dermal, transdermal or ocular drug delivery, they need to be dispersed in a 3D mechanical matrix to remain at the administration site to prevent undesired systemic and local side-effects (irritation, inflammation). The encapsulation of LNP inside a suitable delivery vehicle could prevent such negative features. Indeed, a double encapsulation system—drug loaded into LNP, themselves entrapped in a vehicle material—could provide spatial and temporal control over drug delivery. Moreover, the vehicle, as is the case for certain hydrogels, can display interesting properties such as muco-adhesion and enhanced biocompatibility [31]. The use of a viscous gel formulation is an interesting technical solution, as it often allows more convenient handling of the sample and greater compliance of the patient. Combining lipid nanoparticles and hydrogels is therefore a good solution to achieve better control over the release kinetics of the active molecule. This composite system will display both component advantages: the protection and the improvement of the solubility of the active molecule in lipid nanoparticles, while retaining the good rheological properties of hydrogels and improving the release of the active molecule (Figure 1). 

## 3. Hydrogels as Lipid Nanoparticle Scaffolds

Hydrogels are commonly defined as 3D networks of chemically or physically cross-linked polymers that swell in water while retaining their physical structural integrity. They are hydrophilic, biocompatible and simple to process by different methods to achieve a large variety of 2D (coatings, films) and 3D (bulk gels, aerogels) scaffolds. They also offer a wide range of tailored features by well-known and well-controlled chemical modifications and add the possibility of controlling the drug release spatially and temporally. The degree of cross-linking, the concentration, and the nature of the polymers (either synthetic or natural) are the three key parameters influencing the porosity and the degree of swelling of hydrogels. These two features (porosity and degree of swelling) control the passive diffusion kinetics of active molecules, biomolecules, or nanoparticles loaded inside the matrix [32]. The structure-function relationships of the hydrogel scaffolds play the major part in the drug release behavior. The chemical composition of hydrogel scaffolds can be tuned to respond to an environmental trigger and to degrade after a desired lifetime, allowing researchers to control the gelation process as well as the degradation process. These two possibilities are the key features that determine spatially and temporally targeted drug release. Two main types of drug release mechanisms from hydrogels have been found: diffusive release, based on the second Fick’s law of diffusion; and degradation-based release. 

Two main issues remain unaddressed when hydrogel constitutes the only material of the drug delivery system. Because of the large size of gel pores and their high water content, small and water-soluble drugs quickly escape from the network, leading to a burst release effect and reduced bioavailability. The stable incorporation of hydrophobic drugs within the hydrogel network is also not easy to achieve. Due to the incompatibility between the hydrophobic drug and the water-swollen hydrogel matrix, such molecules tend to precipitate or to rapidly escape from the material [33]. To overcome these limitations, lipid nanoparticles, as presented in the first part of this review, are prime candidates to promote the good solubility of lipophilic entrapped active molecules by the double encapsulation process. 

**Figure 4:**
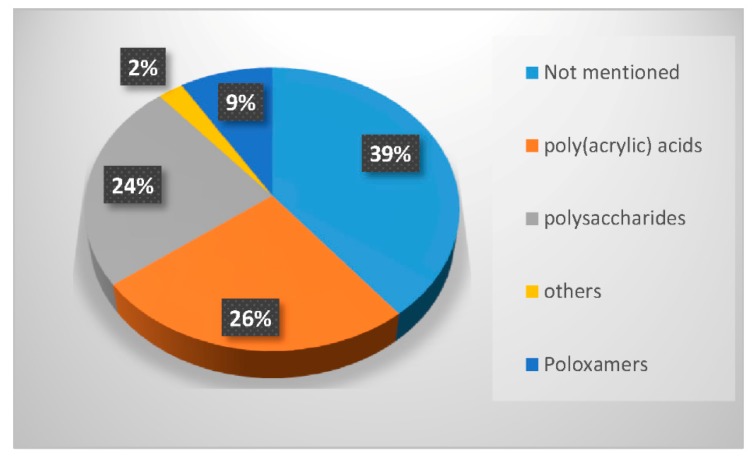
Percentage of occurrence of the different types of polymers used to form hydrogels entrapping LNP in the last twenty years.

In the last twenty years, researchers have used different types of polymers to create hydrogel scaffolds. From synthetic polymers to natural polysaccharides, they all display useful properties. The statistical bibliography analysis performed for this review classifying the LNP-hydrogel hybrid drug delivery systems by polymer type is presented in Figure 4 and Table 1. Results are extracted from bibliography research performed on Scopus with roughly 125 relevant papers. A large majority of LNP-hydrogel drug delivery systems use cheap, commercially available and ready-to-use formulations of poly(acrylic) acids (commonly named Carbopol^®^) (26%). The main component of the hydrogel may not even be specified (39%), as these polymers are mainly used as a spreading vehicle to give the formulation the convenient viscosity for in vitro and in vivo characterizations, and dermal topical delivery. In these cases, the non-specified polymer is also very often revealed to be Carbopol^®^ polymer. As described in Table 1, poly(acrylic) acid hydrogels have a gelation process based on the entanglements of polymer chains. In this case, the synthesis of LNP-hydrogel composites is simple: an aqueous solution containing a determined amount of LNP dispersion and Carbopol^®^ is stirred for homogenization and adjusted to the ideal Carbopol^®^ gelation pH (indicated by the supplier, generally being between 6 and 8), yielding the LNP-hydrogel composite.

However, to face drug delivery challenges imposed by specific physiological conditions, developing smart hydrogels sensitive to changes in their immediate environment has become crucial. Other synthetic polymers, such as poloxamers—thermosensitive synthetic block polymers described in Table 1—were also used (9% occurrence). The synthesis of LNP-poloxamer hydrogel composites is similar to that of LNP-poly(acrylic) acid hydrogel composites, as the gelation process is also based on physical interactions. The mixing of LNP dispersion and poloxamer powder in an aqueous solution is done at low temperatures (around 4 to 10 °C) to avoid early gelation. Once the mixture is homogeneous, it is progressively brought back to room temperature, or a temperature suitable for further characterization and use.

Finally, the biological properties of polysaccharides, especially their very high biocompatibility, have strongly attracted researchers to use them as LNP scaffolds for more advanced systems (24% occurrence). As the gelation process of a majority of polysaccharides requires chemical or physical cross-linking by addition of a suitable cross-linker, the mixing of the LNP dispersion, polysaccharide, and cross-linker is done under appropriate conditions. For example, chitosan needs to be dispersed at acidic pH in aqueous solution. The obtained hydrogel can be neutralized by washing steps at the end of the process. If it is associated with cross-linkers that induce thermo-gelation [34], the mixing step will be carried out at 4 °C. In the case of photo-activated cross-linking reactions [35], LNP dispersion, modified polysaccharides and associated polymers will be homogeneously dispersed in aqueous solution prior to exposition to UV light. Polysaccharides constitute the third-most used polymer family in LNP-hydrogel composite engineering (Figure 4).

Thorough characterizations of LNP-hydrogel materials are still very scarce, certainly due to the recent emergence of this research domain (the first LNP-hydrogel composite was developed in 2004 [11]). Even rheological or mechanical experiments, classically used for controlling hydrogel properties, are not systematically performed for composites, for which shallow description such as “material displays the suitable rheological properties for spreading” can be encountered, without any quantitative value. Drug release kinetics is, however, quasi-systematically quantified in in vitro conditions. Ex vivo experiments, for instance on skin samples mounted on Franz cell set-up, or in vivo experiments in rodent models, are also very scarce. Most advanced and noticeable studies will be detailed in the following sections, organized by hydrogel family.

### 3.1. LNP-Poly(Acrylic) Acid-Based Scaffolds

As demonstrated in Figure 4, 26% of the hydrogels used to entrap lipid nanoparticles are synthetic anionic poly(acrylates) with a high molecular weight, more commonly known under the name Carbopol^®^. Their general chemical structure is described in Table 1. 39% of hydrogel users do not explicitly detail the chemical component of their formulation, but it is often revealed to be a derivative of poly(acrylate) as well. Poly(acrylate) is usually cross-linked with components such as allyl ethers of pentaerythritol, or allyl ethers of sucrose, allowing a very wide range of molecular weights to be commercially available [36,37]. Generally, less than 1% *w*/*v* of Carbopol^®^ powder in solution is necessary to obtain a formulation with the appropriate viscosity for a topical use. When simple hydrogels, not responsive to their surrounding environment, are needed, they provide ready-to-use, cheap and biologically well-tolerated formulations. Despite the hydrogel network being charged due to the acrylate moieties, LNP, being positively or negatively charged, do not interfere with the hydrogel network [13]. The zeta potential of the whole system is governed by the surface charges of the hydrogel [13,38]. The capacity to homogeneously disperse LNP in the pre-hydrogel solution before starting the gelation process ensures an optimized controlled drug release. To formulate a Carbopol^®^ hydrogel, the solid powder needs to be neutralized up to a neutral pH (between 5.7 and 7 [39]) to free the bound alkali ions associated with the carboxylic moieties. Usually, electrolytes such as sodium hydroxide are added. With hybrid LNP-hydrogel systems, however, their use should be avoided, as the alkali ions disrupt the ionic balance, leading to the aggregation of the LNP [13,18]. As an alternative, non-ionic neutralizers can be added, such as triethanolamine or ethylenediamine.

**Figure 5:**
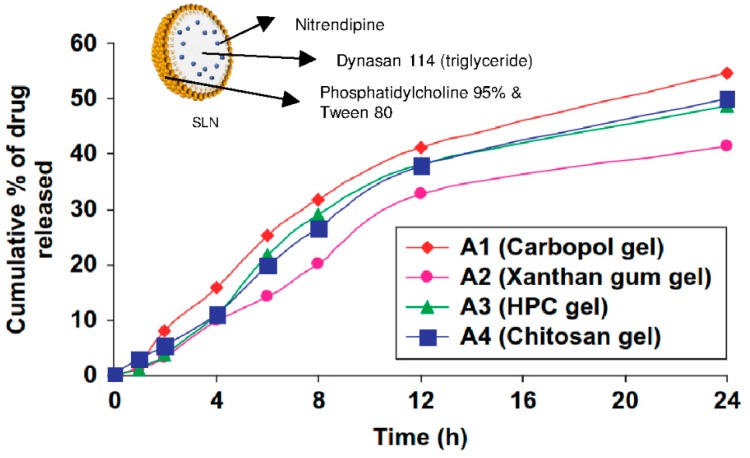
Cumulative time-release profile of nitrendipine-loaded SLN from different hydrogels. Adapted from [13].

In one recent study [13], Carbopol^®^, xanthan, and hydroxypropylcellulose were compared as hydrogel support for nitrendipine-loaded solid lipid nanoparticles and nanostructured lipid carriers. As described earlier, the zeta potential (around −15 mV) and the size of the nanoparticles (around 200 nm) were not influenced by the hydrogel types. As displayed in Figure 5 for SLN, the drug release rate profile was similar for all the hydrogels types, but Carbopol^®^ gels displayed a higher final cumulative release rate of 54% after 24 h for SLN and 64% after 24 h for NLC. Finally, the Carbopol^®^ gel was selected to carry out the in vitro (rat skin permeation) and in vivo tests (pharmacokinetics of nitrendipine and efficacy of hydrogels for treating hypertension in rats) because of its optimum rheological properties and thermal stability [13]. 

Another recent study confirmed the interest of dual systems composed of LNP and Carbopol^®^ hydrogels. Four different dual delivery systems were developed for the sustainable release of voriconazole (a recent anti-fungal agent) [40]. By comparing microemulsions, nanostructured lipid carriers (NLC) and their combination with Carbopol^®^, the authors pointed out the benefits of the hybrid NLC-hydrogel system. The nanostructured lipid carriers were shown to prolong and control the release of the drug and to improve the permeation through the skin, while the Carbopol^®^ hydrogels confirmed their ability to also slow down the release rate of voriconazole. The synergy between NLC and Carbopol^®^ was clearly demonstrated by the higher quantity of drug permeating through the skin up to the dermis with the NLC-hydrogel hybrid system, closely followed by the NLC aqueous dispersion. 

### 3.2. LNP-Poloxamer-Based Scaffolds 

Stimulus-responsive hydrogels have gained much attention in the last ten years. They can be useful in many pharmaceutical applications: targeted and controlled drug delivery systems [41,42], tissue engineering [43], wound dressings [43,44], and smart diagnostic assays [44,45]. Their sol-gel transition can be triggered by several kinds of stimulus: change in pH and temperature [41,42,43,45,46], application of UV light [41,44], and electric or magnetic field [44]. 

Among these, thermo-responsive hydrogels are very interesting for injectable drug delivery systems. In such cases, the gelation process takes place when the environment temperature rises from room temperature to body temperature. Poloxamers are FDA- and EMA-approved triblock copolymers composed of a central hydrophobic poly(propylene) oxide (PPO) unit flanked by hydrophilic poly(ethylene) oxide (PEO) units, as depicted in Table 1. Thanks to their well-characterized thermo-reversible gelation process at specific concentration and temperature, poloxamers have been the most used components for synthesizing biocompatible thermo-sensitive hydrogels. Two main poloxamers are usually found in these compositions: poloxamer 188 (commercialized under the name Pluronic^®^ F68 by BASF) and poloxamer 407 (commercialized under the name Pluronic^®^ F127 by BASF). Their chemical structures are, respectively, PEO_80_-PPO_30_-PEO_80_ and PEO_106_-PPO_70_-PEO_106_.

In this context, thermo-sensitive materials using poloxamers and lipid nanoparticles have been increasingly studied for specifically sensitive delivery routes: injectable [43,47], ocular [47,48] or rectal drug delivery [12]. These drug delivery systems allow a spatially and temporally controlled and sustained drug delivery at the site of application. As previously mentioned, lipid nanoparticles also improve the bioavailability of the encapsulated drug, an effect maintained in the case of the association of LNP and hydrogels. 

Hao et al. developed a thermosensitive hydrogel containing Resina Draconis-loaded solid lipid nanoparticles for ocular drug delivery [47]. Ocular drug delivery is one of the most challenging types of topical drug delivery. The blinking reflex, the rapid tear turnover, the nasolacrimal drainage effect, and the mechanical barrier constituted by the cornea explain the low bioavailability of the drug after delivery into the eyes [34]. Using the central composite factorial method [47], the authors determined the optimal concentrations of poloxamer 407 and poloxamer 188 used for the synthesis of a polymer gelling at the most suitable temperature. The results were 27.8% (*w*/*v*) of poloxamer 407, and 3.55% (*w*/*v*) of poloxamer 188 for a gelling temperature of between 33.4 °C and 35 °C. These physical and biological characterizations gave an insight into the molecular interactions in the drug delivery system. LNP size strongly increased from 150 nm to 450 nm after incorporation into the hydrogel. This important modification could be due to the adsorption and entanglement of the poloxamer 188 chains onto the surface of the lipid nanoparticles. The association of LNP and hydrogels showed promising biological results; the whole system did not trigger any in vivo irritation in the cornea, and improved the trans-corneal delivery of lipophilic drugs [47].

**Figure 6:**
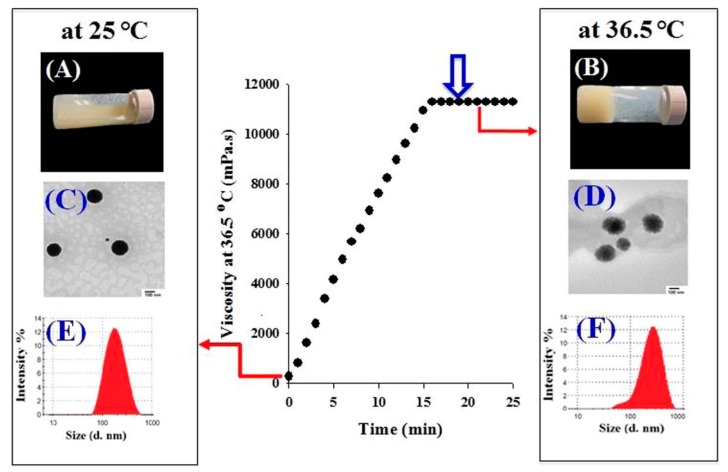
Schematic representation of the irinotecan-loaded SLN-hydrogel system behavior at 25 °C and 36.5 °C. Adapted from [12].

In one recent study, Din et al. used a double thermo-responsive drug delivery system [12]. By associating a mixture of poloxamer 407, poloxamer 188 and Tween 80 with thermo-responsive irinotecan-loaded lipid nanoparticles, they created a system homogeneous at 25 °C, but displaying two phases at 36.5 °C. As displayed in Figure 6, lipid nanoparticles have a neat circular shape and an average hydrodynamic diameter of 178 nm at 25 °C. The hydrogel is still in a liquid state. At 36.5 °C, lipid nanoparticles display larger size, as well as fuzzier edges, on TEM images. LNP seem to be in a liquid state, while the hydrogel has already undergone its sol-gel transition. The system also showed improved pharmacokinetic parameters: the irinotecan release rate was delayed, and the maximal concentration of the released drug decreased, giving a controlled and sustainable drug delivery profile. Irinotecan is a potent and highly toxic anticancer agent; its systemic side effects are harmful for the patient. In this regard, the fact that irinotecan plasma concentration remained at a rather low level longer than all the other tested systems was a very good pharmacokinetics indicator. Moreover, contrary to other drug delivery systems presently available for intramuscular delivery, the in situ gelling did not initiate any irritation or local damage to the surrounding muscular tissues after injection. Poloxamer/irinotecan-LNP therefore appears as a very good tool in the treatment of metastatic colorectal cancers. This system would be a promising sustainable material for intramuscular delivery with reduced side effects.

Synthetic poloxamers, however, display one major drawback when it comes to pharmaceutical applications. They lack adhesiveness, particularly with regard to biological tissues such as skin, nasal or lung mucosa, for instance. Therefore, Lee et al. added to an LNP-poloxamer hydrogel formulation a small amount of xanthan, a biocompatible polysaccharide known for its bio-adhesion properties, as well as its ability to considerably delay drug delivery [49]. In this recent study, curcumin-loaded lipid nanoparticles were added to an aqueous solution of 23% (*w*/*w*) mixture of poloxamer (407 and 188), 0.05% to 0.20% (*w*/*w*) xanthan, and double-distilled water, to create a hydrogel for transdermal drug delivery. At a skin temperature of 32 °C, different amounts of poloxamer 407 (P407) and poloxamer 188 (P188) were tested to obtain a tailored gelling temperature. At a ratio of 10:90 (P407/P188), the hydrogel underwent its sol-gel transition at a bit under 30 °C. The inclusion of curcumin-loaded LPN in poloxamer/xanthan (P/XG) hydrogels did not significantly influence their physical parameters. The authors also compared the drug release behavior and the adhesiveness of the system LNP–P/XG hydrogel and of the system LNP–P hydrogel. Meanwhile, the drug release rate remained close for both hydrogels, the adhesiveness was higher and stronger with the addition of xanthan, as expected. As previously reported in other studies mentioned above [12,47], the association of LNP with the P/XG hydrogel slowed down the release rate of the active ingredient and decreased the maximal concentration of the released drug. The addition of a polysaccharide successfully added biological properties to the transdermal, thermo-sensitive, double drug delivery system. 

### 3.3. LNP-Polysaccharide Based Scaffolds 

In the biomedical field, polysaccharides have sparked the interest of researchers because of their attractive properties [50]. They are composed of long chains of monosaccharide units bound together by glycosidic linkages. Their advantages in the synthesis of hydrogels for medical applications lie in several features. They are naturally abundant, biocompatible, mucoadhesive, biodegradable, have a high affinity toward water, and allow for a wide range of chemical modifications [50,51,52]. Moreover, the chemistry involved in their structural modifications can generally be carried out in mild conditions, which is a great asset for future medical applications. 

**Figure 7:**
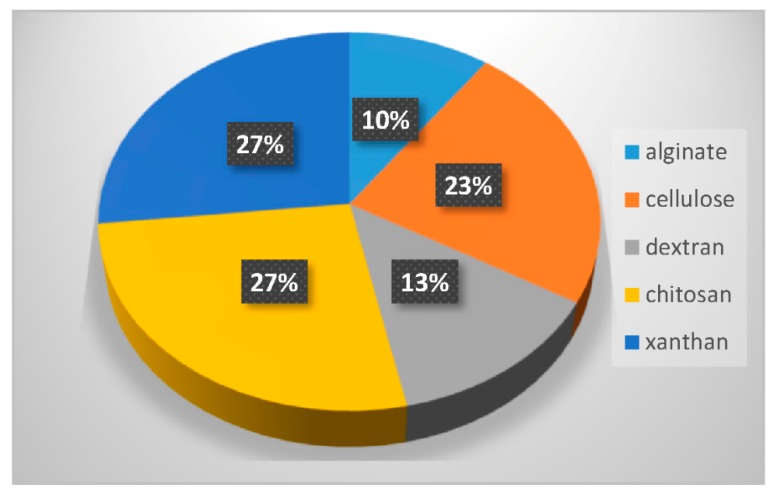
Percentage of the different polysaccharides used in the last 10 years in combination with LNP.

Used in their natural state, polysaccharide hydrogels display rather poor mechanical properties. For example, they can be too brittle or too fragile or even start to degrade at physiological pH. Two strategies can be used to overcome these limitations. They consist in modifying the polysaccharide backbone, or in combining polysaccharides with other synthetic polymers. These two strategies have been used to design hybrid LNP-polysaccharide-based hydrogel systems. Table 2 summarizes different combinations of polysaccharide-based hydrogels and LNP, which are discussed in the following sections. Figure 7 displays the percentage of the different polysaccharides used over the last 10 years in combination with LNP. The statistical bibliography analysis was carried out using the database Scopus, yielding 30 relevant results. The three most used polysaccharides in combination with LNP can thus be highlighted: chitosan (27% of occurrence), cellulose (23%) and xanthan (27%). Dextran (10%) and alginate (10%) are also quite prominently represented. Xanthan was mainly used for its viscosity enhancing properties and generally plays a role in LNP-hydrogel formulation similar to that previously described for poly(acrylic acid) in Section 3.1. It will not be further discussed in this review. The following sections focus on the other different polysaccharides. 

#### 3.3.1. Hydrogel-Lipid Nanoparticle Systems Based on Cellulose Derivatives

Cellulose is the most abundant of natural biopolymers, as it can be found in plants and all kinds of natural fibers. It fulfills two key features that explain its common use in biomedical applications: it is biocompatible and biodegradable. However, due to its structure (Table 2) and numerous hydrogen bonds created between polymer chains, cellulose is highly rigid and semi-crystalline; it is not soluble in water or in most common organic solvents. To overcome these issues, hydroxyl groups from the glucose units of cellulose are generally chemically modified through esterification or etherification [63]. In the pharmaceutical field, the most used cellulose derivatives, also called cellulosics, are hydroxyethylcellulose (HEC), hydroxypropylcellulose (HPC), hydroxypropylmethylcellulose (HPMC), and sodium carboxymethylcellulose (CMCNa) [64]. They are all water-soluble and commercially available, with various molecular weights, degrees of substitution and positions of the substituents. These parameters determine the physico-chemical properties of the cellulose ethers such as their viscosity in solution, their surface activity, their sensitivity to biodegradation and to oxidative stress. 

A first way to control the release profile of a drug from a cellulose-based material is to design the complete drug delivery system based on surface charge interactions. Pure hydrogels of cellulosics were used to entrap lipid nanoparticles loaded with 5-fluorouracil [56] and topotecan [53] (two hydrophilic chemotherapeutic agents active against carcinoma), avanafil [55] (a hydrophobic molecule active in erectile dysfunction) and nano-emulsified copaiba oil [54] (anti-inflammatory essential oil). 

Khallaf and collaborators tested three different hydrogels to introduce 5-Fluorouracil-loaded LNP with a size of 140 nm [56]. They tested chitosan (a positively charged biopolymer), hydroxypropylcellulose (neutral, hydrophilic polymer) and sodium carboxymethylcellulose (negatively charged polymer). The best permeation parameter, defined as the cumulative amount of drug permeated after 24 h per unit area and measured thanks to a modified Franz cell, was obtained with CMCNa. This result was explained by the electrostatic interaction between the positively charged lipid nanoparticles and the negatively charged CMCNa network [56]. In the study of the kinetics of release of avanafil from neutral LNP embedded in hydroxypropylmethylcellulose (HPMC) films, a neutral cellulosic network [55], LNP-loaded HPMC films also showed a better cumulative diffusion of avanafil compared to the LNP-loaded chitosan films after the initial release phase. The release kinetics of LNP from HPMC films followed the Fick’s second law diffusion model and, in this context, neutral HPMC films seemed more adapted to the diffusion of LNP compared to the positively charged chitosan films. 

In another case [54], lipid nanoparticles containing copaiba oil were synthesized with positive and negative surface charges. In this recent study, after testing three different hydrogels (chitosan, Carbopol 980 and hydroxyethylcellulose), HEC hydrogel was the only one tested that did not show any interference in the stability parameters for both positive and negative LNP. The study also showed that the anti-inflammatory effect of the complete system remained successfully equivalent to that of ketoprofen (a well-known non-steroidal anti-inflammatory drug). Venancio and collaborators [53] also pointed out that the neutral charge of HEC films encapsulating topotecan-loaded LNP allowed more of the lipid nanoparticles to be retained in the stratum corneum and in the deeper layers of the skin compared to LNP-loaded chitosan films. Because of its neutral charge, HEC polymer was then proven to have a wide range of application as LNP host. 

A second way to control the release of the drug loaded in LNP-hydrogel systems is to play with the density of the hydrogel network. A hybrid hydrogel was developed by Racine and collaborators [35], thanks to a UV photo-activated thiol-ene cross-linking reaction. Adding a second polymer in the hydrogel network makes easier to precisely modulate the density of cross-links. Alkene-functional CMC and poly(ethylene)-bis(thiol) concentrations were then modified to obtain three different hydrogels with various degrees of cross-linking, defined by the ratio R of the thiol moiety concentration divided by the alkene group concentration (R = [SH]/[=]). Gels with R = 0.2, 0.5 and 1 were formulated. LNP with different surface charges and diameters were then included in the hydrogels. The influence on the release rate of the fluorescent molecule (used as a drug model) of the gel rheological behavior and swelling ratio was tested. The presence of the LNP did not affect the mechanical properties of the gels. Even a high LNP/CMC payload (50% w/w_CMC_) did not influence the gelation kinetics. On the other hand, the particle charge and the concentration of cross-links changed the release kinetics. As expected, the softest hydrogel (R = 0.2) showed the highest release rate. A smaller cross-linking degree led to a looser network, facilitating the diffusion out of the hydrogel for the lipid nanoparticles. The release profile once again followed the Fick’s second law of diffusion model. It was possible to model the behavior of the LNP into the network, taking into account steric hindrance and electrostatic interactions. Aside from the nanoparticle and hydrogel network surface charges, it is then possible to play on the concentration of the hydrogel cross-links to modulate the long-term LNP release rate. 

#### 3.3.2. Hydrogel-Lipid Nanoparticle Systems Based on Chitosan Derivatives

Chitosan possesses unique physico-chemical and biological properties, justifying its common use in biomedical applications [65]. Aside from being highly biocompatible and biodegradable, it possesses hemostatic properties that make it an attractive biopolymer building-block for the design of wound dressings. The specific physico-chemical properties of CS offer many ways of processing it in the form of sponges, films, and hydrogel networks with specific features such as injectability and stimulus-responsiveness [35]. 

However, when used as the main component of the hydrogel network, chitosan also displays some disadvantages for LNP composite hydrogels. First, it is generally accepted that chitosan is insoluble in neutral or alkaline aqueous solutions. The brittleness of chitosan hydrogels is also not adapted to dermal, transdermal or wound dressing applications [57]. 

Moreover, it has already been reported that, in contrast to cellulose derivatives, chitosan influences the LNP parameters after their inclusion into the hydrogel network. Lucca et al. [54] described an increase in the droplet size of lipid nano-emulsions (LNE) from 247 nm to 524 nm, and in polydispersity index (PDI) values from 0.051 to 0.489 after their inclusion in a chitosan gel. Among the different causes, the interactions between the protonated amine group of the chitosan network and the surface charges of LNE may trigger the aggregation of small droplets, explaining the increase in PDI values [54]. In addition, the strong electrostatic interactions between chitosan polar groups and the LNE surface charge prevent most of LNE from crossing the first skin barrier [53], explaining why less LNE remains on the stratum corneum after material application compared to the cellulosic-based LNE-hydrogel formulation [54]. However, LNE have been found in deeper layers of the skin when LNE-loaded chitosan hydrogels were used in comparison to cellulosic-based hydrogels. The polar groups of the chitosan might be able to open the tight junction between membranes of deeper skin layers, leading to further penetration of some LNE. 

**Figure 8:**
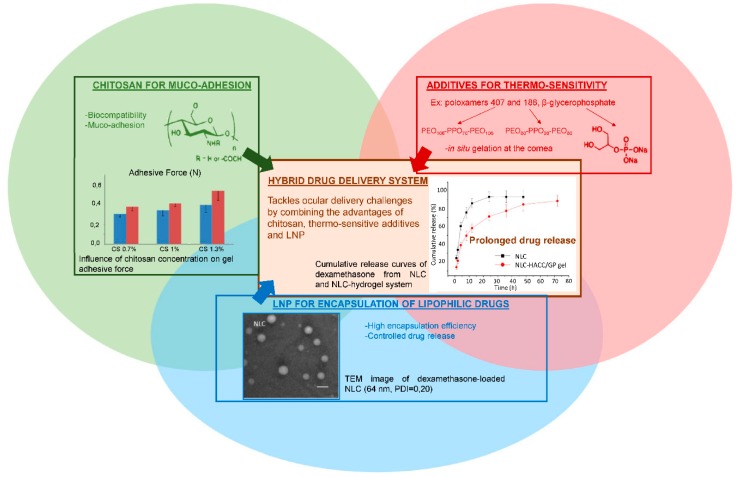
Advantages of combining several components to formulate an innovative drug delivery system that meets ocular administration route expectations. Adapted from [14,34,54].

As described earlier, topical drug delivery into eyes is challenging. Thermo-sensitive synthetic polymers such as poloxamer 407 display interesting in situ gelling properties, improving the pre-corneal residence time of the drug without creating irritation. As already mentioned, one main drawback of such synthetic polymers is that they lack bio-interactions. In contrast, chitosan is a good candidate for ocular delivery formulations, since it displays very interesting bio-adhesion properties. The amino groups of the chitosan network are known to form secondary chemical bonds (hydrogen bonds or electrostatic interactions) with the negatively charged sialic acid moieties of mucins [14]. Therefore, one of the main solutions suggested to prolong the residence time of the drug in the eyes is to use thermo-sensitive chitosan-based hydrogels. By combining chitosan with a synthetic block copolymer such as poloxamer 188 [14,58] or with β-glycerophosphate [34], the hybrid system retained the biological properties of chitosan while being thermo-responsive, as shown in Figure 8. In a liquid state at room temperature, these gels underwent a sol-gel transition at a tunable temperature to become a semi-solid system. Almeida and collaborators demonstrated that a hybrid drug delivery system of ibuprofen-loaded LNP in a 1% chitosan and 20% of poloxamer 407 dispersion was suitable for ocular drug delivery [14]. The system displayed appropriate rheological and mucoadhesive properties at pH = 7.4, minimal influence on LNP parameters, respect for the physiological limits of particle size and zeta potential before causing ocular irritation, no burst release effect (only 25% of ibuprofen released after 3 h), and a prolonged pre-corneal residence time. 

Modification of chitosan backbone can also be carried out, either to bring new properties to the drug delivery system, such as pH sensitivity, or to further improve the biological properties. In this respect, two chitosan derivatives have been used in combination with the same thermo-sensitive poloxamer 407. Carboxymethyl chitosan (CMCS) is a water-soluble chitosan derivative that exhibits pH sensitivity in addition to biocompatibility and bio-adhesion. Mixing CMCS with poloxamer 407 and cross-linking the whole mixture with genipin [58] created a hybrid hydrogel displaying pH and thermo-sensitivity. The resulting network successfully integrated quercetin-loaded NLC to form a biocompatible, smart and sustainable system designed for ocular drug delivery. After 72 h under physiological conditions (i.e., at 35 °C and pH = 7.4), 20% of the encapsulated quercetin remained in the hydrogel [58]. Hydroxypropyltrimethyl ammonium chloride chitosan (HAAC) is a partially quaternized water-soluble chitosan derivative used by Tan and collaborators to synthesize a thermogel undergoing sol-gel transition in situ at 35 °C, in combination with β-glycerophosphate [34]. Thanks to its cationic quaternary amino group, HAAC is able to strongly bind to the negatively charged surface of the cornea. It is thus viewed as a promising permeation enhancer for the paracellular way of absorption. Dexamethasone, a potent glucosteroid anti-inflammatory agent, was successfully entrapped within lipid nanoparticles embedded in this thermogel [34]. This thermo-sensitive gel then appeared suitable for ocular drug delivery under physiological conditions. 

A novel 3D scaffold integrating nanostructured lipid carriers was used recently [57]. Synthesized from hyaluronic acid and chitosan, these highly porous sponges showed a controlled but fast release of the encapsulated drug. Andrographolide (a wound-healing agent called AND, for short) loaded in NLC was totally released after 48 h at physiological pH. Interestingly, the presence of NLC in the sponge network slightly decreased its swelling behavior after 1, 4 and 7 days. H-bonding and hydrophobic interactions between the NLC coating surfactant, polyoxyethylene 20 cetyl ether, and the chitosan structure could be at the source of this phenomenon. The wound-healing activity of the AND-NLC-scaffolds was enhanced when compared to the controls (NLC dispersion and empty scaffold) [57]. The HA-chitosan scaffold alone displayed a certain anti-inflammatory and antioxidant activity, explaining the better results when both NLC and hydrogels components were joined together in the final system [57]. 

#### 3.3.3. Hydrogel-Lipid Nanoparticle Systems Based on Alginate Derivatives

Alginate is a naturally occurring anionic polysaccharide extracted from brown algae. It is a co-polymer composed of 1-4-linked β-d-mannuronic acid and α-l-guluronic acid units that undergoes a conformation change in the presence of divalent cations to quickly form a gel [51]. Alginate hydrogel is commonly used in pharmaceutical applications because of its biocompatibility, its easy processing and the low costs of producing gel beads. In this regard, alginate seems to be a good candidate for encapsulating lipid nanoparticles in gel beads.

One of the first challenges to address is to evaluate the integrity of LNP after their encapsulation in the calcium alginate beads. In addition to size measurements, few techniques give information about the state of lipid nanoparticles within alginate beads. Differential Scanning Calorimetry (DSC) was used by Strasdat and collaborators to measure the influence of several bead production methods (electrostatic droplet generation method or spraying method) and processes (freezing and lyophilization) on the structure and quantity of lipids constituting the core of LNP (with a diameter < 100 nm) embedded in alginate capsules (with a diameter from 45 µm to 1.3 mm). They concluded that the electrostatic droplet generation method was mainly harmless to the LNP, producing alginate beads from circa 300 µm to 1000 µm. Smaller alginate beads of 45 µm were possible to obtain thanks to the spraying method. However, this method was found to degrade the LNP melting pattern. The shear forces applied during the production process, as well as the drying process, must put an important strain on LNP structure, altering the lipid state. When it comes to the purification step, freezing the LNP-loaded alginate beads is possible with the addition of a sufficient amount of cryogenic protector. It is also not a surprise that lyophilization intensely altered the state of the incorporated lipids [62]. 

One of the key features of these gel beads is their sensitivity to pH. At low pH, the hydrogel swelling ratio is low, with the hydrogel keeping its structural integrity. At a pH of around 7, the hydrogel starts to swell and to degrade [61]. This particularity was exploited by Senna and collaborators to encapsulate LNP in calcium alginate beads for oral drug delivery [61]. The resistance of the calcium alginate beads at low pH enabled the protection of the encapsulated LNP through the very aggressive environment of the stomach. Then, the increase in the hydrogel swelling ratio due to the higher intestinal pH caused the release of the beads’ content in the intestinal tract, where it could best be absorbed by the body. The influence of the alginate mass ratio and the calcium concentration of gelling solutions was studied. Densifying the hydrogel network by increasing the alginate mass and the concentration of the cross-linking agent improved the pH sensitivity of the hydrogel beads [61].

#### 3.3.4. Hydrogel-Lipid Nanoparticle Systems Based on Dextran Derivatives

In contrast to the other widely used polysaccharides, such as alginate, chitosan or hyaluronic acid, dextran does not possess chemical functional groups allowing easy further chemical modifications. On the other hand, it displays excellent biocompatibility. Once modified, it is possible to use it for a wide range of applications. For instance, dextran can act as a promoter of epithelial cell adhesion, or as an anti-inflammatory molecule [66,67]. One of the first examples of a drug delivery composite system composed of solid lipid nanoparticles (SLN) and hydrogels was developed with methacrylate-modified dextran (dextran-MA) [23]. UV photo-activation of the methacrylate moieties of the dextran allowed the formation of the hydrogel in a short time, entrapping the SLN already dispersed in the aqueous solution within the polymeric network. The process of formation of the hydrogel did not damage the structure of the SLN. After 2 h of incubation in a 0.1 M HCl solution, simulating gastric transit, only 40% of the ibuprofen encapsulated in the SLN had been released. On the contrary, other systems (ibuprofen-dextran-MA, ibuprofen-loaded SLN) typically showed burst release profiles [23]. This example constitutes one of the first occurrences of a composite system, with SLN entrapped in hydrogel compatible with a controlled release of the active molecule in an oral formulation [23]. Later on, the system was tested with a more sensitive anti-fungal molecule called ketoconazole [59]. The complete system still showed promising results during the characterization of the release profile, as well as satisfactory stability of the drug itself. Because this molecule was known to be influenced by environmental conditions [68], Paolicelli and collaborators tested its efficiency at different pH. The whole loaded SLN-dextran system was as effective as the commercial cream (Nizoral) at pH = 5 and pH = 7, displaying its ability to retain the activity of the encapsulated molecule. This system was also able to be modified to obtain the right rheological properties needed for a convenient use from the patient. Tailoring the derivatization degree and dextran concentration could easily change between a semi-solid consistency, a good spreadability, or a rigid hydrogel that remained at its site of application [60]. Densifying the hydrogel network by increasing the polymer concentration and the derivatization degree did not influence the release rate of the ibuprofen, the drug still being much smaller that the size of the meshes [59].

## 4. Conclusions

Different LNP-hydrogel hybrid materials have been designed and studied as drug delivery systems for dermal, transdermal, ocular, intramuscular and transmucosal delivery routes. The combination of LNP and hydrogels has given promising results, as hybrid materials combine the properties of both types of drug delivery system. The biocompatibility, the dramatically improved stability of the encapsulated drug, and the possibility of precisely controlling the drug release kinetics, both spatially and temporally, make these hybrid systems versatile, tunable and suitable for a wide range of applications. New findings on the analytical methods for evaluating LNP stability have allowed researchers to gain a better understanding of the interactions between the nanocarriers and hydrogel networks, leading to better control of drug release. These successful formulations can specifically improve the ocular, dermal, and oral routes, which are particularly challenging.

In recent years, emphasis has been placed on the development of smart hydrogels, able to respond to an external stimulus such as pH changes, temperature changes, or mechanical triggers. They not only act as a scaffold for LNP aqueous dispersion, but also play an active role in the spatial and temporal control of drug release. For example, poloxamers have been extensively studied for their thermo-responsive properties. In this context, polysaccharide-based hydrogels have shown exceptional properties. Specifically, their high biocompatibility and their simple and easy-to-control chemical modifications make them attractive building blocks for the development of such hydrogels. The combination of several polysaccharides or the combination of polysaccharides with synthetic polymers has yielded the design of original hybrid systems, able to critically tune the drug release. 

Despite the significant progress made in the last two decades, further studies are still to be carried out on the thorough characterization of these hybrid systems. Mechanisms of drug release control are still not very well understood and need further investigation. Mechanical properties are not yet systematically evaluated, rendering system performance comparison not always appropriate and the overall outlook of such technology shallow. The wide range of features that smart polysaccharide-based hydrogels offer is yet to be fully explored, and this type of material would benefit from further research. 

Where the clinical potential of such LNP-hydrogel drug delivery systems has been demonstrated with in vitro trials of numerous types, there is a lack of uniformity in the drug release, mechanical and biological characterizations of such LNP-hydrogel systems. This heterogeneity is the reason comparison between various hybrid LNP-hydrogel system efficiency is not currently relevant. This technology is still in its early infancy (the first LNP-hydrogel composite was developed in 2004 [11]), and very few hybrid systems have reached further than in vitro testing, explaining why there is still a large gap between academic research studies and the use of these promising materials in clinics. However, the interesting properties and advanced functionalities of LNP-hydrogel hybrid systems should yield to the development of innovative drug delivery solutions in dermal, transdermal, ocular and oral delivery routes in the future. The latest advances in ocular delivery, as developed in Section 3.3.2, are particularly encouraging. In this context, it will be of capital interest in the future to collect more data on the efficiency and long-term effects of these materials in the frame of preclinical and then clinical studies.

## Figures and Tables

**Table 1:** Types of hydrogels used for LNP-hydrogel combination in the past 5 years.

Polymer Family	Chemical Structure	Type of Chain Interactions	Properties
Poly(acrylic) acid polymers (ex: Carbopol^®^)	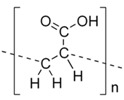	Entangled chains	Easy-to-use rheological properties and biocompatibility
Poloxamers (ex: Pluronic^®^)	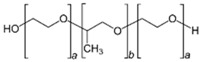	Physical gels	Thermo-sensitivity and biocompatibility
Polysaccharides (ex: cellulose, alginate, …)		Mainly chemical cross-linking	Bioactivity and biocompatibility

**Table 2:** Combination of LNP and polysaccharide-based hydrogels in the past 5 years.

Polysaccharide	Associated Polymer	Type of LNP/Drug	References
Hydroxyethylcellulose 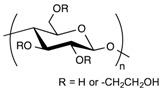		NLC (≈120 nm) // Topotecan LNE (≈280 nm) // Copaiba oil	[53,54]
Hydroxypropylmethylcellulose 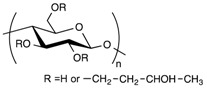		SLN (<100 nm) // Avanafil	[55]
Carboxymethylcellulose 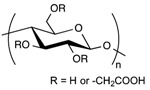		SLN (≈140 nm) // 5-Fluorouracil	[56]
	PEG	NLC (50 nm and 120 nm) // DiI	[35]
Chitosan 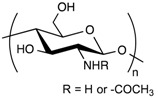		SLN (≈200 nm) // Nitrendipine LNE (≈280 nm) // Copaiba oil NLC (≈120 nm) // Topotecan SLN (<100 nm) // avanafil	[13,53,54,55]
	Pluronic^®^ F127	NLC (≈150 nm) // Ibuprofen	[14]
	Hyaluronic acid	Sponge scaffold // Andrographolide	[57]
Hydroxpropyltrimethyl ammonium chloride chitosan 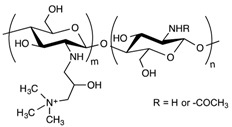	β glycerophoshate	NLC (≈65 nm) // Dexamethasone	[34]
Carboxymethyl chitosan 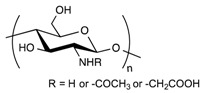	Pluronic^®^ F127	NLC (≈75 nm) // Quercetin	[58]
Dextran 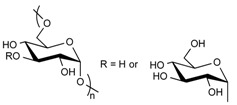		SLN (130–270 nm) // Ketoconazole	[23,59,60]
Alginate hydrogel 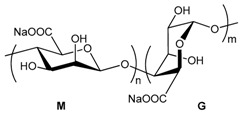		NLC (≈85 nm) // Amphotericin B	[61]
Alginate beads 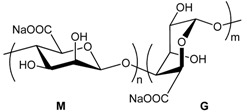		LNE (<100 nm) // Ø	[62]
